# Cannabinoids in Chronic Pain: Therapeutic Potential Through Microglia Modulation

**DOI:** 10.3389/fncir.2021.816747

**Published:** 2022-01-07

**Authors:** Nynke J. van den Hoogen, Erika K. Harding, Chloé E. D. Davidson, Tuan Trang

**Affiliations:** ^1^Department of Physiology and Pharmacology, University of Calgary, Calgary, AB, Canada; ^2^Department of Comparative Biology and Experimental Medicine, University of Calgary, Calgary, AB, Canada; ^3^Hotchkiss Brain Institute, University of Calgary, Calgary, AB, Canada

**Keywords:** microglia, chronic pain, cannabinoid, analgesia, nociceptive circuitry

## Abstract

Chronic pain is a complex sensory, cognitive, and emotional experience that imposes a great personal, psychological, and socioeconomic burden on patients. An estimated 1.5 billion people worldwide are afflicted with chronic pain, which is often difficult to treat and may be resistant to the potent pain-relieving effects of opioid analgesics. Attention has therefore focused on advancing new pain therapies directed at the cannabinoid system because of its key role in pain modulation. Endocannabinoids and exogenous cannabinoids exert their actions primarily through G_i/o_-protein coupled cannabinoid CB1 and CB2 receptors expressed throughout the nervous system. CB1 receptors are found at key nodes along the pain pathway and their activity gates both the sensory and affective components of pain. CB2 receptors are typically expressed at low levels on microglia, astrocytes, and peripheral immune cells. In chronic pain states, there is a marked increase in CB2 expression which modulates the activity of these central and peripheral immune cells with important consequences for the surrounding pain circuitry. Growing evidence indicate that interventions targeting CB1 or CB2 receptors improve pain outcomes in a variety of preclinical pain models. In this mini-review, we will highlight recent advances in understanding how cannabinoids modulate microglia function and its implications for cannabinoid-mediated analgesia, focusing on microglia-neuron interactions within the spinal nociceptive circuitry.

## Introduction

Acute nociceptive pain functions as an alarm system that protects us from serious injury. Chronic pain, however, can be likened to a dysfunctional alarm system: it may persist for months and years after an injury has healed. Silencing this alarm can be difficult, or paradoxically, it may sound even in the absence of injury. One in five people worldwide are affected by chronic pain, which is characterized by spontaneous pain, allodynia (a painful experience of a non-painful stimuli), and hyperalgesia (an increased pain experience to a painful stimuli). Converging evidence indicates that chronic pain arises because of maladaptive plasticity resulting in sensitization of nociceptive circuitry within the peripheral and central nervous system (CNS). Overall, these adaptations shift the balance between excitatory and inhibitory signals in the spinal dorsal horn, a hub for nociceptive processing, toward excitation (Coull et al., 2005; Knabl et al., 2008; Liu et al., 2008; Bonin and De Koninck, 2014; Hildebrand et al., 2016). This shift can result in aberrant amplification of sensory input and output from the spinal cord, which gives rise to exaggerated pain responses that underlie chronic pain. The increase in nociceptive drive is causally linked to a constellation of cellular and molecular processes that alter neuronal excitability and fundamentally change activity of glia, such as microglia and astrocytes (Tsuda et al., 2005; Zhuang et al., 2005; Hains and Waxman, 2006; Sorge et al., 2015; Nam et al., 2016; Kohro et al., 2020; reviewed in Tsuda, 2016; Ji et al., 2019). In this mini-review, we will focus on microglia, immune cells that reside within the CNS and account for 5–20% of the total cell population (Lawson et al., 1990; Perry, 1998). Microglia are exquisitely sensitive to perturbations of the nervous system, responding to injury, disease, or infection. A feature of this microglia response is increased reactivity, which is associated with a plethora of changes in receptors, intracellular proteins, and transcription factors that a causally implicated in the development and maintenance of chronic pain (Chen et al., 2018b; Inoue and Tsuda, 2018).

Effective management of chronic pain remains one of the most difficult clinical challenges, as it is often refractory to conventional therapies like opioids (Dellemijn, 1999; Kalso et al., 2004; O'connor and Dworkin, 2009). To manage pain, some people turn to cannabis and cannabinoid products, making pain the most common medical reason for cannabis use (Mucke et al., 2018; Voelker, 2018; Boehnke et al., 2019; Solomon and Solomon, 2019). The efficacy of cannabis in treating chronic pain is contentious and often stigmatized. Indeed, the International Association for the Study of Pain (IASP) does not currently endorse the use of cannabis or cannabinoids for pain relief purposes (IASP Presidential Task Force on Cannabis, 2021). However, there is evidence suggesting cannabis may provide some relief from cancer pain and neuropathic pain (Andreae et al., 2015; Whiting et al., 2015; Mucke et al., 2018; Wang et al., 2020). Most medical cannabis products are phytocannabinoids, which are derived from plants of the *cannabis* genus. While over 60 active compounds can be found in cannabis plants, phytocannabinoids (CBs) like Δ-9-tetrahydrocannabinol (THC) and cannabidiol (CBD) are the most well studied. These compounds typically act upon the endogenous cannabinoid receptors type 1 (CB1R) and type 2 (CB2R). CB1Rs are found at key nodes along the pain pathway, and their activity modulates both the sensory and affective components of pain, while CB2Rs are expressed (albeit typically at low levels) on immune cells (Pertwee, 2001; Guindon and Hohmann, 2009). Both the CB1R and CB2R are G_i/o_-coupled and inhibitory, with activation resulting in the inhibition of calcium channels through G protein signaling, activation of potassium channels, and the inhibition of adenylate cyclase activity (Howlett et al., 2002; Guindon and Hohmann, 2009). Among the most well characterized endogenous CB receptor ligands are anandamide (AEA) and 2-arachidonoyl-glycerol (2-AG). AEA is a high-affinity, partial agonist of the CB1R and CB2R (reviewed in Stella, 2010; Biringer, 2021). 2-AG has moderate affinity for both CB receptors as a full agonist. The endogenous cannabinoid system also encompasses their synthesizing enzymes, N-acyl-phosphatidylethanolamine-phospholipase (NAPE-PLD) and diacylglycerol lipase (DAGL), and metabolic enzymes, fatty acid amide hydrolase (FAAH) and monoacylglycerol lipase (MAGL) (Lu and Mackie, 2021). Endocannabinoids regulate diverse brain functions like memory, feeding, reward, neuroprotection, neural development, sleep, and pain (Lu and Mackie, 2021). Notably, activation of CB1R and CB2R in the spinal dorsal horn modulates nociceptive transmission (Finn et al., 2021; Soliman et al., 2021). CB1R expression has been detected on spinal primary and secondary afferents, and on astrocytes (Hohmann et al., 1998, 1999). By contrast, CB2Rs are mostly expressed on cells of the immune system and upregulated in inflammatory or disease states, including chronic pain (Zhang et al., 2003; Walczak et al., 2006; Romero-Sandoval and Eisenach, 2007; Racz et al., 2008b; Brownjohn and Ashton, 2012; Naguib et al., 2012; Shiue et al., 2017). Both CB1R and CB2R agonists have been shown to attenuate pain behaviors in a variety of animal pain models (for a comprehensive review of preclinical studies, see Finn et al., 2021; Soliman et al., 2021). In addition, CB2Rs are immunomodulatory and their activation decreases neuroinflammatory responses in a microglia-dependent manner (Lu and Mackie, 2021), making it a potential target for refractory pain.

## Spinal Microglia: Contributors to Chronic Pain

**Figure 1:**
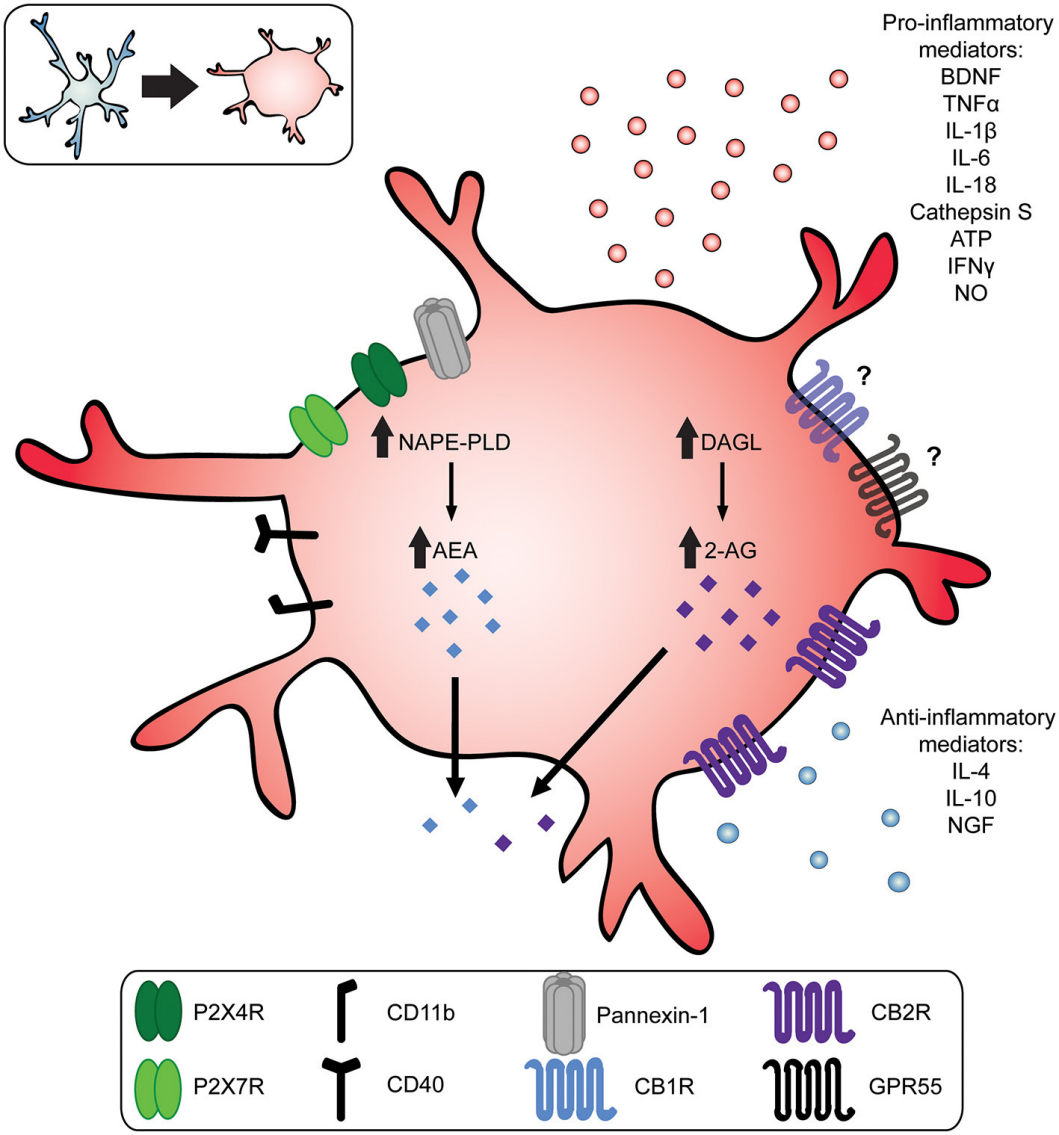
Reactive microglia increase production of endocannabinoids and pro-inflammatory mediators. In response to injury, infection, or pathology of the nervous system, microglia transition toward a more reactive phenotype. This transition is characterized by a morphological change from a small soma with long, ramified processes (top left, blue) to a more amoeboid shape (top left and center, red). Upon activation, microglia release pro-inflammatory mediators, such as brain-derived neurotrophic factor (BDNF); adenosine triphosphate (ATP); nitric oxide (NO), interleukin 1β (IL-1β), IL-6, IL-18, cathepsin S, interferon γ (IFNγ), and tumor necrosis factor α (TNF-α). Reactive microglia also increase production of the endocannabinoids anandamide (AEA) and 2-Arachidonoylglycerol (2-AG) mediated by N-acyl-phosphatidylethanolamine-phospholipase (NAPE-PLD) and diacylglycerol lipase (DAGL), respectively. Endocannabinoids and exogenous cannabinoids act on microglia CB2Rs, which are upregulated in chronic pain states, or they may act on CB1Rs or GPR55. This in turn upregulates the release of anti-inflammatory cytokines like interleukin 4 (IL-4); IL-10; and nerve growth factor (NGF).

Microglia are resident immune cells of the CNS with diverse functions, including the maintenance and pruning of synapses, providing trophic support of neurons, glutamate uptake, and phagocytosis of extracellular debris (Latremoliere and Woolf, 2009; Burke et al., 2016; Harte et al., 2018). Under homeostatic conditions, microglia possess a highly ramified morphology characterized by a small soma and long branching processes that survey the microenvironment (Nimmerjahn et al., 2005). In response to injury or disease of the CNS, microglia shift toward a reactive phenotype that releases proinflammatory cytokines, chemokines, and other immune mediators (Inoue and Tsuda, 2018). This microglia-mediated neuroimmune response critically contributes to chronic pain (Coull et al., 2005; Hains and Waxman, 2006; Beggs et al., 2012; Echeverry et al., 2017; Chen et al., 2018a). Several mechanisms are known to drive microglia reactivity observed in neuropathic and inflammatory pain conditions, including the activation of CSF1, P2X4, and P2X7 receptors (Tsuda et al., 2003; Chessell et al., 2005; Guan et al., 2016). For example, nerve injury resulting in mechanical allodynia gives rise to reactive spinal microglia characterized by the *de novo* synthesis of P2X4 receptors (Tsuda et al., 2003; Trang et al., 2009; Mapplebeck et al., 2018). ATP activation of P2X4R+ reactive microglia evokes the release of brain derived neurotropic factor (BDNF), which in turn downregulates the potassium-chloride cotransporter 2 (KCC2) in spinal laminae I/II neurons (Beggs et al., 2012). The disruption of chloride homeostasis results in enhanced excitation and diminished inhibition of spinal nociceptive output, implicated in the aberrant mechanical pain sensitivity following nerve injury (Coull et al., 2005; Mapplebeck et al., 2019; Ferrini et al., 2020). In addition, reactive microglia act through P2X7 receptors, the production of nitric oxide (NO), and the release of pro-inflammatory cytokines like tumor necrosis factor α (TNFα), interleukin-1β (IL-1β), and cathepsin S (CTSS) (Clark et al., 2007; Guan et al., 2016; Kobayashi et al., 2016; Kanda et al., 2017; Dalgarno et al., 2018; Mousseau et al., 2018). Thus, a myriad of microglia mechanisms involving diverse signaling pathways and pro-inflammatory cytokines (Figure 1) contribute to central sensitization underlying the aberrant nociceptive processing associated with chronic pain (Coull et al., 2005; Kawasaki et al., 2008).

## Microglial CB2R in Nociceptive Signaling

Converging evidence indicates that CB2Rs play an important role in regulating chronic pain states, although their exact expression pattern has been a topic of intense debate (Rogers, 2015). Resting microglia typically express low levels of CB2R (Duffy et al., 2021), but this expression is markedly increased in inflammatory and neuropathic pain states (Zhang et al., 2003; Walczak et al., 2006; Romero-Sandoval and Eisenach, 2007; Racz et al., 2008b; Brownjohn and Ashton, 2012; Naguib et al., 2012; Shiue et al., 2017; Grenier et al., 2021). The extent of this CB2R increase appears to be dependent on the pain model used (Zhang et al., 2003) and may promote microglia to switch toward a more anti-inflammatory phenotype (Bie et al., 2018; Komorowska-Muller and Schmole, 2020). Studies employing CB2R agonists (e.g., JWH-015, JWH-133, HU-308, or AM1241) report marked antinociception in a variety of preclinical pain models, including spinal transection (Romero-Sandoval et al., 2008), postsurgical pain (Romero-Sandoval and Eisenach, 2007; Grenald et al., 2017), sciatic nerve injury (Beltramo et al., 2006; Cabanero et al., 2020), bone cancer pain (Lu et al., 2015; Wang et al., 2020), chronic constriction injury (Niu et al., 2017), and formalin (Hanus et al., 1999; Grenald et al., 2017). However, pharmacologically discerning the specific contribution of CB2Rs has been difficult because many CB2R agonists also have activity on CB1Rs, which are more abundantly expressed. Specifically, JWH-015, JWH-133, and AM1241 are between 26 and 200 times more selective for CB2R compared to CB1R, but these agonists can still activate CB1Rs. Thus, conclusions about CB2R involvement in these chronic pain models have been strengthened by using CB2R antagonists such as SR144528 or AM630 (Hanus et al., 1999; Beltramo et al., 2006; Naguib et al., 2008; Romero-Sandoval et al., 2008; Lu et al., 2015). Furthermore, studies using global CB2R knockout or myeloid specific CB2R knockout found increased hyperalgesia and allodynia in sciatic nerve injury models (Racz et al., 2008a,b; La Porta et al., 2013; Nent et al., 2019), while overexpression in hematopoietic cells, including microglia, leads to an overall decrease in pain behaviors (Racz et al., 2008b). These experiments also demonstrate that CB2R activation restrict microglia activity to the ipsilateral dorsal horn, as knockout of the CB2R leads to a spread of hypersensitivity and microgliosis to the contralateral dorsal horn after sciatic nerve injury or in arthritis induced by monoiodoacetate (Racz et al., 2008a; La Porta et al., 2013; Nent et al., 2019). Double knockout of CB2R and interferon gamma (IFN-γ) blunted the spread of hypersensitivity, suggesting the effect is mediated by the pro-inflammatory cytokine (Racz et al., 2008a).

**Figure 2:**
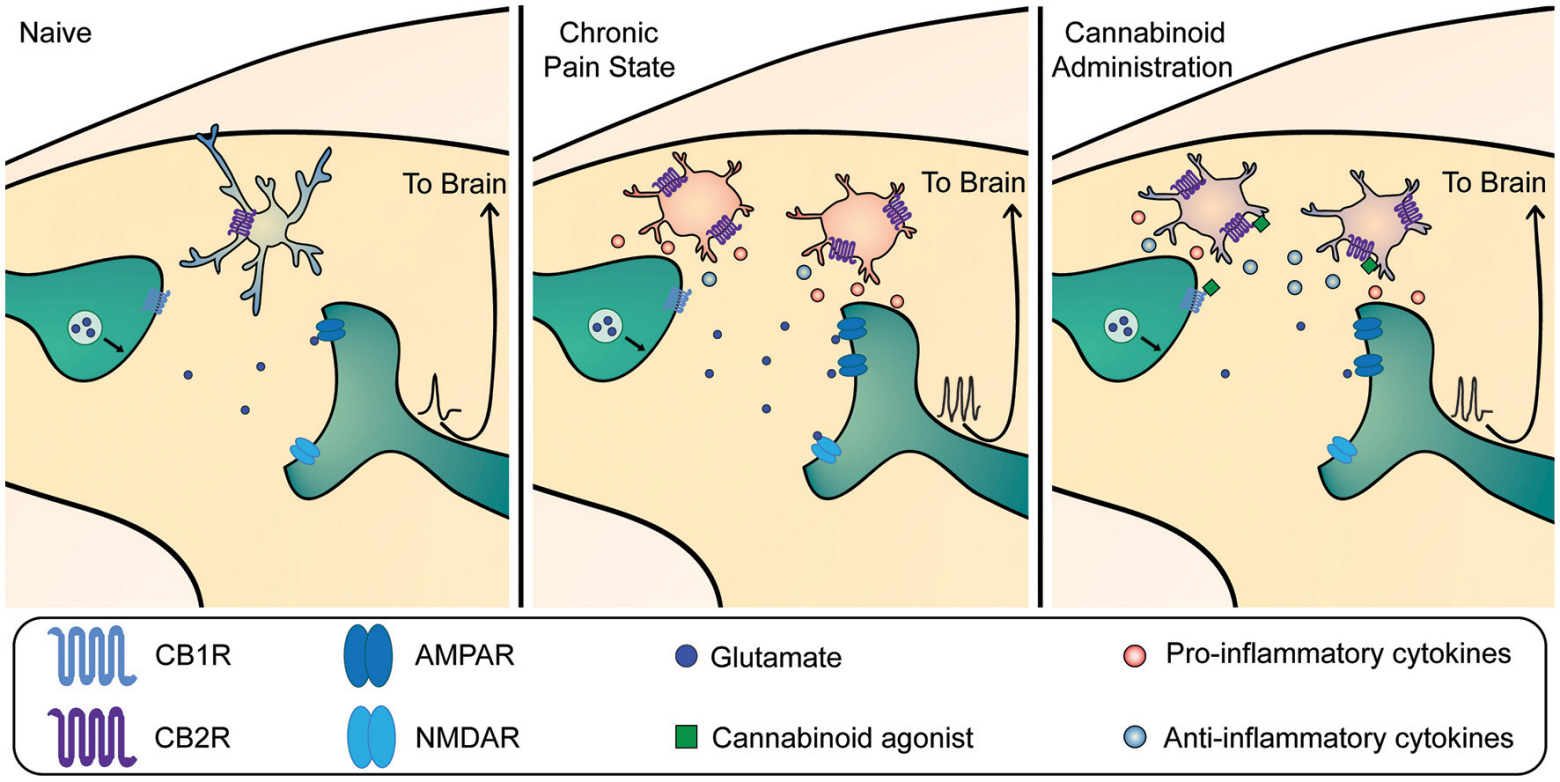
Mechanism of cannabinoid-mediated reduction in dorsal horn hyperexcitability in chronic pain. Left panel: Under homeostatic conditions, microglia are present in their surveillant phenotype. Cannabinoid receptor 1 (CB1R) is expressed on primary afferent terminals. Mid panel: In chronic pain states, microglia become active and contribute to dorsal horn neuron hyperexcitability through release of pro-inflammatory mediators that act on nociceptive circuitry to increase output sent to the brain. This occurs in tandem with other neuronal mechanisms including an increase in α-amino-3-hydroxy-5-methyl-4-isoxazolepropionic acid receptor (AMPAR) trafficking and phosphorylation, and phosphorylation of N-methyl-D-aspartate receptors (NMDAR), which all contribute to central sensitization. Right panel: Exogenous and endogenous cannabinoids can bind to CBRs on both neurons and microglia within the dorsal horn. CB1Rs are present predominantly on neurons at the presynaptic terminal, where their activation decreases vesicular release to reduce glutamate release onto nociceptive projection neurons. CB2Rs are present predominantly on microglia, where their activation shifts microglia to produce more anti-inflammatory mediators and fewer pro-inflammatory mediators. Together, activation of both neuronal and microglial CBRs leads to a reduction in nociceptive output.

Overall, there is compelling evidence that activation of CB2R critically modulates microglial immune function by switching to a more anti-inflammatory state, characterized by limited migration (Walter et al., 2003; Eljaschewitsch et al., 2006), phagocytosis (Tolon et al., 2009), increased production of anti-inflammatory mediators, and decreased production of pro-inflammatory mediators (Figure 2) (Ehrhart et al., 2005; Ashton and Glass, 2007; Racz et al., 2008a; Malek et al., 2015; Ma et al., 2021). Specifically, the activation of CB2R on microglia, either through the action of endogenous cannabinoid AEA, or exogenous cannabinoids like THC and CB2R agonists, has been shown to inhibit the production and release of pro-inflammatory molecules causally involved in central sensitization (Eljaschewitsch et al., 2006; Correa et al., 2009). For example, activation of the CB2R (i) upregulates the AMP kinase pathway to decrease synthesis of nitric oxide (Giri et al., 2004), (ii) downregulates the p38 MAP kinase pathway to reduce synthesis of IL-1β, TNF-α and BDNF (Ji and Suter, 2007; Niu et al., 2017), and (iii) suppresses ERK-mediated microglia proliferation (Zhuang et al., 2005; Calvo et al., 2011; Naguib et al., 2012). Indeed, CB2R activation in a paclitaxel model of chemotherapy-induced neuropathy leads to decreased IL-6, BDNF, P2X4, and TNFα receptor expression, and increased release of the anti-inflammatory cytokine IL-10 (Burgos et al., 2012; Wu et al., 2019). This cannabinoid-induced release of IL-10 by microglia is a phenomenon shared with other areas of the CNS (Correa et al., 2010; Hernangómez et al., 2012). Additionally, activation of CB2R by exercise-induced AEA release reduces levels of TNFα and IL-1β in the spinal cord of mice with carrageenan-induced pain hypersensitivity (dos Santos et al., 2019). In these studies, the reduced release of pro-inflammatory cytokines and increased release of anti-inflammatory cytokines by microglia was associated with reduced pain behavior.

Another contributor to microglial-dependent central sensitization is the activation of microglial purinergic receptors. Specifically, P2X4 and P2X7 receptor activation is linked to development of several chronic pain conditions (Tsuda et al., 2003; Trang et al., 2006; Ulmann et al., 2008; Beggs et al., 2012; Sorge et al., 2012; Masuda et al., 2016), albeit in a sexually dimorphic manner (Sorge et al., 2015; Mapplebeck et al., 2018). Treatment with a CB2R agonist reduces P2X4 upregulation in paclitaxel-induced neuropathy (Wu et al., 2019), providing another mechanism through which CB2R activation can reduce microglial contributions to pain.

## Microglial Production of AEA and 2-AG

Microglia not only express CB2Rs and respond to cannabinoid agonists, but they also possess the cellular machinery to produce endocannabinoids (Carrier et al., 2004; Walter and Stella, 2004; Witting et al., 2004). Remarkably, microglia may be the main endogenous source of endocannabinoids, as they can produce up to 20-times more endocannabinoids than other glial cells or neurons *in vitro* (Walter et al., 2003). The synthesis of AEA and 2-AG is dependent on an increase in intracellular calcium levels (Di Marzo, 2008). In microglia, this increase in calcium may be achieved by activation of purinergic receptors such as P2X4 and P2X7 (Witting et al., 2004; Di Marzo, 2008). Endocannabinoids are highly lipophilic molecules and as a result they are released soon after production (Alger and Kim, 2011). AEA binds preferentially to CB1R *in vitro*, but also displays a low binding affinity for transient receptor potential cation channel subfamily V member 1 (TRPV1), which is highly expressed in the nociceptive system (Zygmunt et al., 1999; Storozhuk and Zholos, 2018). 2-AG activates both CB1R and CB2R. Spinal sensory neurons mainly express CB1R, although there is evidence for neuronal CB2R expression in chronic pain states (Wotherspoon et al., 2005). In these neurons, CB1R activation reduces presynaptic vesicular release into the spinal dorsal horn through reduction of calcium entry via Cav2.2 and potassium efflux through KCC2 (Howlett et al., 2004; Zamponi, 2016). In addition, binding of (endogenous) cannabinoids at the spinal level inhibits the activity of adenylyl cyclase, preventing the conversion of ATP to cAMP, thereby reducing nociceptive signaling to higher order neurons. Moreover, endocannabinoids reduce nociceptive signaling by directly inhibiting serotonergic receptor 5-HT3 (Fan, 1995; Barann et al., 2002) and reducing sodium influx (Nicholson et al., 2003), and endogenous cannabinoids such as AEA may directly block the voltage-gated calcium channel Cav3.2, located at the presynaptic terminal between primary afferents and the superficial dorsal horn (Chemin et al., 2001; Barbara et al., 2009; Zamponi, 2016).

Under neuropathic pain conditions, activated spinal microglia increase their production of AEA and 2-AG significantly by upregulating NAPE-PLD and DAGL, while degrading enzyme FAAH is downregulated (see Figure 1) (Eljaschewitsch et al., 2006; Garcia-Ovejero et al., 2009; Mecha et al., 2015). The increased release of endogenous cannabinoids in turn drives CB1R and CB2R activity to dampen nociceptive signaling. In addition, 2-AG and AEA upregulates the expression of microglial CB2R, and possibly CB1R, promoting a neuroprotective phenotype. Although reactive microglia are considered proinflammatory and exacerbate pain phenotypes, the increase in endocannabinoid release and upregulation of CB2Rs appear to be compensatory mechanisms that keep microglia reactivity in-check and reduce neuronal hyperexcitation.

## Role of Microglial CB1R and Other Cannabinoid Receptors in Nociceptive Signaling

The localization of CB1R vs. CB2R has been a major debate fueled by limitations in available tools to discern anatomical and cell specific expression profiles. Early studies relied on antibody approaches to map CBR expression, but it was later determined that these antibodies may lack specificity (Atwood and Mackie, 2010; Baek et al., 2013; Zhang et al., 2019). To overcome this problem, groups have developed highly selective agonists and antagonists directed against the CB1R and CB2R, as well as engineered a variety of transgenic mice targeting key nodes within the endocannabinoid system (Glass and Northup, 1999; Griffin et al., 1999). Sequencing studies from single cells and tissues have also provided important insights into the localization of CBRs (Carlisle et al., 2002; Pietr et al., 2009). These technological advances suggest that in addition to CB2R, microglia may express other CB receptors. For example, CB1Rs are found throughout the CNS and primarily on neurons and astrocytes, but low levels of CB1R mRNA have also been reported in cultured microglia from rodent brain cortex (Waksman et al., 1999; Stella, 2010). Activation of these receptors by CP55940 blocks NO release after microglial activation using endotoxin *E. coli* lipopolysaccharide (LPS). This effect was blocked by CB1R antagonist SR141716, suggesting CB1R expression on microglia (Waksman et al., 1999). However, the presence of CB1R expression has not been found in human microglia to date (Stella, 2010; Mecha et al., 2016). Microglia also appear to express CB receptors that are neither CB1R nor CB2R. For example, CB receptor agonist levonantradol reduces LPS-induced expression of proinflammatory cytokines like IL-1α and TNF-α, an effect that was not reversed by blocking CB1R and CB2R with selective antagonists SR141716A and SR144528 (Puffenbarger et al., 2000). Similarly, treatment with cannabinoid agonist WIN55212-2 inhibited TNF-α release in a CB1R and CB2R independent manner (Facchinetti et al., 2003). These results suggest the presence of non-classical CBRs in microglia that may also be involved in the cannabinoid modulation of pro-inflammatory protein expression. Indeed, a G-protein coupled receptor GPR55 expressed on microglia has been shown to be activated by cannabinoid agonists, HU-210, G405833, and L-α-lysophosphatidylinositol (LPI) (Oka et al., 2007; Ryberg et al., 2007; Henstridge et al., 2010; Anavi-Goffer et al., 2012). Interestingly, treatment with CB1R inverse agonist SR141716 has produced opposing effects on GPR55 activity, with some studies reporting an activation (Henstridge et al., 2010) while others demonstrating an inhibition of the receptor (Lauckner et al., 2008). Adding further to this puzzle, CBD appears to suppress GPR55 activity (Whyte et al., 2009). Recently, the fatty acid ethanolamide palmitoylethanolamide (PEA) has gained interest because of its anti-inflammatory and antinociceptive properties (Re et al., 2007; Guida et al., 2017). While it does not bind to CB1R or CB2R, PEA may directly or indirectly stimulate CB2 and CB1 receptors (Re et al., 2007; Lin et al., 2015; Guida et al., 2017), and its anti-inflammatory actions are blocked by selective CB2 antagonist SR144528 (Iannotti et al., 2016). Interestingly, PEA increases CB2R expression by activating PPAR-alpha and thereby induces microglial phagocytosis and migration, providing another feedback loop by which cannabimimetic compounds engage the endocannabinoid system to modulate neuropathic pain (Franklin et al., 2003; Guida et al., 2017). These findings collectively point to the complexity of CBR pharmacology and open the possibility that microglia activity may be modulated by a complement of classical and non-classical CBRs with diverse signaling pathways.

## Conclusions and Future Perspectives

Many studies, using a variety of rodent pain models, point to the analgesic efficacy of cannabinoids and their impact on microglia function. There has been particular focus on CB2Rs, which are markedly increased in spinal microglia in chronic pain states, and selective activation of these receptors dampens microglia reactivity and alleviates pain hypersensitivity. Attention has therefore shifted to developing CB2R directed therapies to mitigate the psychoactive side-effects associated with CB1R strategies. However, the effects of CB2R may not be entirely dictated by its actions on the central nervous system or solely by its inhibition of microglia reactivity. For instance, peripheral nerve injury can also induce CB2R expression in rat sensory neurons and a variety of immune cells (Wotherspoon et al., 2005). Indeed, there are important hurdles to overcome to translate CB2R discoveries into realized pain therapies. Notably, there is the question of whether microglial activation patterns (and upregulation of CB2R expression) are differentially impacted in the various chronic pain states. In addition, it will be important to determine whether the side effects of cannabinoid use in a clinical setting can be decreased, while maintaining the analgesic effect. Finally, unraveling the interactions between CB1R and CB2R expression and function, as well as the interplay between neurons, microglia, and astrocytes, will be critical for harnessing the full potential of cannabinoids as a possible treatment for chronic pain and for discerning whether different pain modalities will be more responsive than others. Recent technological advances in vape delivery of phytocannabinoids, and the availability of human microglia cultures from induced pluripotent stem cells, provide new tools to study the full potential of cannabinoids in treating chronic pain and its impact on microglia.

## Author Contributions

NvdH wrote the first draft of the manuscript. EH designed the figures. NvdH, EH, and TT wrote sections of the manuscript. TT provided supervision. All authors contributed to manuscript revision, read, and approved of the submitted version.

## Funding

This work was supported by an Alberta Innovates mCANNABIS grant to TT. NvdH was funded by an Alberta Innovates Postgraduate Fellowship, EH was funded by an Eyes High University of Calgary Fellowship, and CD was funded by a Cumming School of Medicine studentship.

## Conflict of Interest

TT is co-founder and CEO of AphioTx Inc., which is developing pannexin-1 channel targeted therapies. There is no overlap or conflict of interest as it relates to the contents of this manuscript. The remaining authors declare that the research was conducted in the absence of any commercial or financial relationships that could be construed as a potential conflict of interest.

## Publisher's Note

All claims expressed in this article are solely those of the authors and do not necessarily represent those of their affiliated organizations, or those of the publisher, the editors and the reviewers. Any product that may be evaluated in this article, or claim that may be made by its manufacturer, is not guaranteed or endorsed by the publisher.
